# On the Treatment of Chronic Inflammation of the Bladder by Injections of Nitrate of Silver

**Published:** 1847-10

**Authors:** 


### ECLECTIC DEPARTMENT,

### AND SPIRIT OF THE MEDICAL PERIODICAL PRESS.

---

*On the treatment of Chronic Inflammation of the bladder by injections of nitrate of silver.* By ROBERT L. MACDONNELL, M. D. Licentiate of the King and Queen's College of Physicians, and of the Royal College of Surgeons, Ireland; Lecturer on the Institutes of Medicine, M'Gill College; Physician to the Montreal General Hospital, &c.

I believe it will be generally admitted, that of all diseases, few occasion more anxiety and embarrassment to the medical practitioner, or are productive of more misery and suffering to the afflicted patient, than chronic inflammation of the bladder; the conseqence of previous disease of the urinary apparatus, as gonorrhœa or stricture, or as a primary affection produced by other and more general causes.

In either case, when once fully established, it entails the most excruciating suffering on its victim, prevents him following his usual avocations, debars him from society, and not unfrequently produces death, either from the morbid action extending to the ureters and kidneys, from ulceration corroding through the walls of the viscus, or from the exhaustion of the system it is certain to induce.

In a disease of such importance, it is only to be expected that numerous remedies should have been proposed for its cure, and consequently we find agents of various natures strongly recommended by practical writers, yet they all admit the difficulty attending the treatment of the malady, and the failure that has followed the use of their favorite remedies.

Some years ago, Mons. Lallemand, the eminent professor of Montpellier, discovered, accidentally, the great value of nitrate of silver in chronic inflammation of the bladder, and the utility of this plan was shown in a paper by Dr. O'Bryen, in the fourteenth volume of the Dublin Medical Journal. But this gentleman does not appear to have had any personal experience of its employment, and moreover, he confines his remarks to use of the solid nitrate, the form preferred by Lallemand.

I am not aware that he has done more in this matter than introduce to the notice of British surgeons the views of the distinguished professor.

When a student of the Richmond Surgical Hospital, Dublin, I had an opportunity of seeing my friend and former preceptor, Dr. Hutton, (to whom I am indebted for much information that I have found valuable in my profession,) inject the bladder with a weak solution of nitrate of silver, but as this had (if I recollect rightly) but little effect upon the disease, the practice was abandoned in the hospital, nor have I since either seen it employed or heard of its being employed by any one but myself, and I can find no mention of it in those valuable records of medical practice—Braithwaite's Retrospect, and Ranking's Digest, or in the recently published Systems of Surgery.

Having met with some cases of chronic cystitis, that resisted general treatment, and bearing in mind the great success which attended the application of nitrate of silver in substance, in the hands of Lallemand, I determined to give the remedy a further trial in the form of solution, and the success I met with, has far surpassed my most sanguine expectations; I have now no hesitation in stating, that as far as pure uncomplicated chronic inflammation of the bladder is concerned, that the opprobrium has been removed from surgery, and that we do possess a method of treatment followed by a greater amount of success than usually attends remedies employed in diseases of so severe and intractable a nature, and infinitely greater than attends the use of any remedy in a disease hitherto considered by the first authorities as incurable.

In proof of this assertion, I shall adduce four cases, two of which occurred in my private practice, and the other two were witnessed in the wards of the Montreal General Hospital, by a large and intelligent class. I could adduce others, but these I bring forward sufficiently support the views I am anxious to inculcate.

Case I.—A genntleman cousulted me last February, under the following circumstances:—He had suffered for some months from inflammation of the bladder, marked by frequent desire to pass water, accompanied by heat and scalding, violent straining, pain in the region of the bladder, above the pubis and in the perinœum, and a constant feeling of heat and weight in the lower portion of the abdomen. There symptoms gradually increased in severity. The urine became at first bloody, and afterwards purulent, and the desire to void it became so urgent, that it had to be yielded to, at least every fifteen minutes; the discharge of the fluid being followed by pain and scalding at the neck of the bladder, and along the course of the urethra. His general health became impaired, and his sleep being so frequently disturbed, a haggard and anxious expression of countenance, and extreme irritability of the system, were soon established.

When he first consulted me, fully one-half of the fluid passed from the bladder was pure pus; and after repose, a deposit of blood globules was found to intervene between this and the supernatant urine—the latter being highly alkaline, fœtid, and albuminous. Examined microscopically, it exhibited some scales of nucleated epithelium, a large deposit of triple phosphate in prismatic crystals, pus, and blood globules. There was no pain in the loins or along the ureters. He had a stricture of long standing, about one inch from the orifice of the urethra. In addition to the above characters, the urine was frequently mixed with tenacious masses of lymph varying in length from half an inch to an inch, and entangling a quantity of earthly matter, they frequently obstructed the passage of the urine through the stricture, and required to be broken up and squeezed through by the pressure of the patient's fingers.

Having dilated the stricture, so as to allow a large sized catheter (No. 11 Weiss) to pass, I determined to treat the disease by injections of nitrate of silver; and accordingly, on 17th of February, I injected into the bladder a lotion composed of eight grains of lunar caustic, two drachms of tincture of hyosciamus, and four ounces of distilled water.

The injection caused hardly any inconvenience, except that of inducing a strong desire to empty the bladder, which was prevented by compressing the penis, until the fluid had been in the bladder for about one minute, when it was allowed to escape. The next day, the patient stated that he was somewhat better, but the quantity of pus and blood was not, however, much diminished, and the flakes of lymph were more numerous and larger than before. Although he continued improving, yet, as the amendment was not as rapid as I anticipated, injection of the viscus was again resorted to on the 5th of March. On this occasion, the quantity of caustic was increased to sixteen grains in the four ounces of distilled water, and the hyosciamus was omitted. A decided improvement immediately followed; the frequency of making water was greatly diminished; instead of requiring to be voided every fifteen minutes, the bladder could retain its contents for more than two hours at a time, and the quantity of pus had greatly decreased. An injection, of the same strength, was again employed on the 28th of March, and with happy results. The urine could now be retained for three or four hours, was passed without pain or scalding, was clear and transparent, and, to the naked eye, free from pus; but, when examined microscopically, a deposit of pus globules and some epithelial scales were perceptible. On the 18th of April, I repeated the injection, and since then he has been completely free from any symptoms of his troublesome disease; he has resumed his former mode of life and pursuits, and has been subject to various changes of temperature whilst travelling, without experiencing the least return of his former symptoms.

I should mention that, pending the treatment for the cystitis, he had a severe attack of laryngitis œdematosa, which was near proving fatal.

Case II.—Mr. ——, aged 33, consulted me, July 22, 1847. He stated that four years ago, after exposure to severe cold, he began to suffer from difficulty in making water, and from pain referred to the region of the bladder, and extending along the urethra. At first, he was not obliged to empty the bladder more frequently than usual, and the urine was unmixed with either pus, or blood. About three years ago, however, the symptoms just mentioned having existed for twelve months, he began to suffer from a constant and severe pain at the neck of the bladder, above the pubis, and in the perinæum, greatly increased by pressure and exercise. These symptoms have been gradually increasing in severity up to the present, although he has been for four years under the treatment of different practitioners in this city. He now suffers from a constant burning sensation in the situations just referred to, and an excruciating pain in passing water, which he is obliged to do every fifteen minutes; the quantity voided on each occasion not exceeding a teaspoonful.

On examination, the urine was found to be abundantly mixed with pus, (in an eight ounce phial there wns an ounce and a half of pus and mucus,) with large quantity of blood globules, highly ammoniacal in odour, albuminous, and on being subjected to a microscopical examination, it presented an abundant deposit of triple phosphate in prisms, and epithelium.

In this case, I commenced at once with an injection of sixteen grains of nitrate of silver in four ounces of distiled water. The immediate effects were, the disappearance of the pain which had been constantly present for three years; the urine was passed without any heat, scalding, or uneasiness; and the necessity for emptying the bladder became less frequent; the quantity of pus was much diminished, and no more blood was observed in the deposit, and his nights were passed in ease and comfort.

About a fortnight after, the bladder was again injected with the same quantity of the solution of nitrate of silver, and the improvement which followed was equally remarkable. The urine can now, August 27, be retained for nearly, the usual length of time; it contains barely a trace of pus, and is voided without the slightest pain.

His nights are spent in comfort, his strength has greatly increased, and he has gained flesh. Finding himself so much improved, he has gone to the country for change of air, to expedite his cure. Even should some of the symptoms return, owing to the suspension of the treatment, I have no doubt they will quickly disappear after a third injection of the caustic is had recourse to.

Case III.—A man, aged 26, a laborer, was admitted into the Montreal General Hospital, laboring under paralysis of the lower extremities, the result of a severe injury. In addition, it was discovered that he had lost the power of emptying the bladder, and that the urine was mixed with a quantity of tenacious fœtid mucus and pus.

He remained in Hospital for some time before he came under my care, and then the following was the condition in which I found him:—Loss of motion and sensation of both lower extremities; inability to empty the bladder completely, but yet not requiring the catheter; the urine was constantly dribbing away, when he assumed the erect posture, was highly offensive, mixed with a large quantity of pus, mucus and blood, aud crystals of triple phosphate. It is unnecessary to detail the particulars of the treatment employed for the restoration of the power and sensation of the limbs. Suffice it to say, that after some time, the sensation was completely restored, and he had acquired sufficient power over the limbs to enable him to walk about the wards, but no improvement was observed in the character of the urine. The notes taken by one of my pupils, state, that "the urine was half pus, and caused great pain and scalding in passing."

*January* 3.—He was ordered the following mixture:—℞. Infus. Buchu ℥ vss. Tinct. Buchu ℥ i. Bals. Copaibæ, Liquor Potassæ, Tinct. Hyosciam *aa* ℥ ss—one ounce three times a day.

*Jan.* 7.—The quantity of pus had diminished to about one-third, and he was directed to continue the use of the medicine.

*Jan.* 21.—As the quantity of pus had not perceptibly decreased since last report, I determined to employ injections of nitrate of silver, and as the disease had received a notable check from the internal remedy, I did not consider it necessary to use a stronger solution than one grain to the ounce.

*Jan.* 22.—The urine was much clearer, and the deposit of pus was less by one half than previous to the injection, and he could retain the urine for two hours.

*Jan.* 28.—The bladder was again injected, and next day no deposit was exhibited, and the urine was almost as clear as natural.

This man, soon after the last report was taken, was attacked with maculated typhus, and passed through the disease without suffering the slightest inconvenience from the affection of the bladder; and throughout, the urine exhibited a healthy character, even when examined microscopically.

The case I am now about to detail, I have already published in a paper "On the Use of the Microscope," in this Journal, and I shall now introduce it as if then appeared:—

CASE IV.—A strong, healthy man, aged 20, who had been under the care of my colleague, Dr. Hall, in the Montreal General Hospital, for gonorrhœa, and was discharged cured of the complaint, came to me about a month after his dismissal from hospital, complaining of frequent desire to make water, and of pain and difficulty in doing so. As there was no discharge whatever from the urethra, I thought it advisable to pass a catheter, and not meeting with any obstruction, I collected the urine drawn off by it, and examined it at the moment. It was slightly acid, spec. grav. 1024, at temp. 72° Fahr., coagulated on addition of nitric acid, and yielded an abundant exhibition of pus globules on examination with the microscope. Having no symptoms referrible to disease of the kidneys, I treated him for cystitis, and with decided benefit at first, but as he had not a comfortable residence, and was obliged to walk a great distance to my house, in the late hot weather, I recommended him to enter the General Hospital under my care. Here I had frequent opportunities of directing the attention of the students to his case. The urine being again examined, exhibited not only a deposit of pus globules, but also of blood globules. Notwithstanding this unfavorable complication he was discharged about five weeks after admission perfectly cured.

In this case I injected nitrate of silver solution into the bladder; the quantity of pus immediately diminished, and after the third injection, completely disappeared. The microscope was of the greatest aid to me in every stage of this interesting case.

I introduce this case for the purpose of showing that the injection of a solution of nitrate of silver into the bladder, is not only of use in cases which could have been cured by other means, but that it is eminently successful in those instances which have resisted the most valuable general remedies.

In the foregoing observations, I have made no mention of the method of injecting the bladder which I have found most efficient. I will now make a few practical remarks on the operation:—The patient being placed either in the erect position or on a sofa, a gum elastic catheter, about the size of No. 9 or 10 (Weiss,) is introduced, and water at the temperature of 98° Fahr., is injected through this into the bladder, by means of a caoutchouc bag, or what I prefer, a syringe, with a "three-way valve," by which the fluid can be drawn back from the cavity of necessary. After the bladder has been completely cleansed of any fœtid urine and mucus which may be contained in it, the solution of the caustic, being heated to the same degree, is to be introduced in a similar manner, and allowed to remain there for about one minute, care being taken by compressing the urethra, to prevent its being forcibly ejected by the violent straining that is certain to be induced. The quantity of water or solution should never exceed four ounces, for though the bladder in its healthy state is capable of containing nearly a pint and a half of urine, without being over distened, yet as the quantity it is capable of retaining in severe chronic inflammation seldom exceeds a few tablespoonfuls, the bladder accommodates itself to its diminished contents and gradually becomes smaller, and consequently a large injection would act injuriously in two ways—by over-distending the organ, or by passing up into the ureters. In fact we find it unnecessary to use a larger quantity of the solution than I have mentioned, for it requires some address to introduce even that amount without resorting to force. The patient is then ordered a warm bath, and should the urine become bloody or mixed with shreddy concretions, he should use frequent fomentations and anodynes. But these symptoms seldom last for more than a few hours, and our patient should always be informed that such consequences are likely to be the immediate effects of the operation.

My patients have not suffered from retention of urine, which it appears frequently follows the use of the solid nitrate in the practice of Lallemand, nor have they had any inconvenience which was not readily allayed by an opiate.

The advantages which I consider the solution of nitrate of silver possesses over that substance in a solid form are, First, that we can employ it of various strengths, from one to four grains, or even stronger if necessary. Secondly, we are certain that the application comes in contact with the entire diseased surface. Thirdly, we are also satisfied that it does not act more violently on one part than another. Fourthly, it is more readily employed by an inexperienced operator; and above all, in cannot possibly be attended with any risk, from the apprehension of which, it is not easy to divest the mind, when using the *porte caustique* of Lallemand, and together with the above advantages, it has this also to recomend it, that it will be found at least equally successful.

The foregoing observations apply altogether to *severe chronic inflammation of the bladder;* acute inflammation and mild cases of chronic catarrh of that organ, are manageable by other means, to which it is unnecessary to allude.

In the treatment of these cases, we derive great assistance from the microscope, for long after the naked eye fails to detect pus or blood in the urine, these fluids are recognizable by the microscope, and so long as they are present, our treatment should not be relaxed.

I have intentionally avoided alluding to the general treatment of chronic cystitis, and have omitted some other practical points, to which I shall probably direct attention at some future period.

51, Great St. James' Street, Montreal.

[*From the British American Journal of Med. and Phys. Science.*

